# In vivo multimodal retinal imaging of disease-related pigmentary changes in retinal pigment epithelium

**DOI:** 10.1038/s41598-021-95320-z

**Published:** 2021-08-10

**Authors:** Ratheesh K. Meleppat, Kaitryn E. Ronning, Sarah J. Karlen, Marie E. Burns, Edward N. Pugh, Robert J. Zawadzki

**Affiliations:** 1 UC Davis Eyepod Imaging Laboratory, University of California Davis, Davis, CA 95616 USA; 2 Department of Cell Biology and Human Anatomy, University of California Davis, Davis, CA 95616 USA; 3 Center for Neuroscience, University of California Davis, Davis, CA 95618 USA; 4 Department of Ophthalmology and Vision Science, University of California Davis, Sacramento, CA USA

**Keywords:** Preclinical research, Biomarkers, Experimental models of disease, Imaging and sensing, Fluorescence spectroscopy, Biomedical engineering, Confocal microscopy

## Abstract

Melanosomes, lipofuscin, and melanolipofuscin are the three principal types of pigmented granules found in retinal pigment epithelium (RPE) cells. Changes in the density of melanosomes and lipofuscin in RPE cells are considered hallmarks of various retinal diseases, including Stargardt disease and age-related macular degeneration (AMD). Herein, we report the potential of an in vivo multimodal imaging technique based on directional back-scattering and short-wavelength fundus autofluorescence (SW-FAF) to study disease-related changes in the density of melanosomes and lipofuscin granules in RPE cells. Changes in the concentration of these granules in *Abca4*^−/−^ mice (a model of Stargardt disease) relative to age-matched wild-type (WT) controls were investigated. Directional optical coherence tomography (dOCT) was used to assess melanosome density in vivo, whereas the autofluorescence (AF) images and emission spectra acquired with a spectrometer-integrated scanning laser ophthalmoscope (SLO) were used to characterize lipofuscin and melanolipofuscin granules in the same RPE region. Subcellular-resolution ex vivo imaging using confocal fluorescence microscopy and electron microscopy was performed on the same tissue region to visualize and quantify melanosomes, lipofuscin, and melanolipofuscin granules. Comparisons between in vivo and ex vivo results confirmed an increased concentration of lipofuscin granules and decreased concentration of melanosomes in the RPE of *Abca4*^−/−^ mice, and provided an explanation for the differences in fluorescence and directionality of RPE scattering observed in vivo between the two mouse strains.

## Introduction

The retinal pigment epithelium (RPE) is a monolayer of pigmented cells located posterior to the photoreceptors of the neurosensory retina. The RPE performs critical metabolic and transport functions essential for neuronal homeostasis, and RPE dysfunction leads to photoreceptor degeneration and contributes to a variety of retinal diseases, including age-related macular degeneration (AMD)^1,2^. Therefore, the ability to perform noninvasive, in vivo evaluation of the RPE is of great interest to clinical ophthalmology and ophthalmic research. Further, since pigmented granules in the RPE, such as lipofuscin and melanosomes, undergo morphological and biochemical changes with age and disease, they are ideal candidates to serve as biomarkers for aging and progression of diseases in the outer retina.

Lipofuscin is a generic term given to autofluorescent lipopigment aggregates composed of lipids, metals, and misfolded proteins that accumulate during aging in multiple organs throughout the body^3–5^. In the RPE, these aggregates, termed lipofuscin granules, are composed primarily of indigestible retinoid-conjugated proteins and lipids originating in photoreceptor outer segments^6–10^. In healthy photoreceptors, the protein ABCA4 flips the all-trans-retinal-PE complex to the outer leaflet of the disk membrane, allowing the retinal to be hydrolyzed, reduced, and removed^11,12^. In contrast, A2-PE, a doubly-conjugated complex of two all-trans retinals with PE, cannot be removed from the inner leaflet of the discs by ABCA4, and only exits the outer segment during disc shedding and RPE phagocytosis^11,13^. In aging and/or diseased photoreceptors, indigestible retinoid byproducts accumulate more rapidly than normal^14,15^.The accumulated indigestible byproducts collect in RPE lipofuscin granules and exhibit characteristic autofluorescence from the constituent bisretinoid compounds, including A2E and all-trans-retinal dimers^16,17^. RPE lipofuscin granules are implicated in pathological mechanisms involved in several vision-threatening diseases including age-related macular degeneration and Stargardt disease^18,19^. A hallmark of Stargardt disease is the accelerated accumulation of lipofuscin due to defects in the ABCA4 transporter^20–22^. Similar excessive accumulation of lipofuscin is found in Abca4 knockout (*Abca4*^−/−^) mice, which have been widely used as a model for the preclinical investigation of Stargardt disease^22–24^.

Melanosomes are organelles in which melanin is synthesized and stored in pigmented cells. In the RPE, melanosomes are formed during embryonic development and mature during early postnatal stages, while lipofuscin and melanolipofuscin, which are a mixture of the two precursor granules, accumulate as a function of age. Melanosomes serve to protect the RPE from light damage and light-generated oxygen reactive species^25,26^. Melanosomes have been observed to undergo morphological and photophysical alterations with age, possibly due to photooxidation, decreasing their antioxidation potential^27–30^. The density of RPE melanosomes is also reported to decline with increased lipofuscin load caused by age or disease, where the melanosomes tend to fuse with lipofuscin granules forming melanolipofuscin^6,10,28^. Thus, in vivo monitoring of the changes of melanosomes and lipofuscin in the RPE is of prominent clinical interest.

Over the last decade, optical coherence tomography (OCT) has emerged as a gold standard for clinical retinal diagnostics due to its unique ability to provide high contrast, depth-resolved morphology of the retinal layers in vivo with micrometer resolution^31,32^. Clinical OCT systems use near-infrared light (centered at ~ 850 nm or ~ 1050 nm) to detect the back-scattered light from the retinal layers to generate cross-sectional images. Back-scattered light from the retinal layers can also provide critical information regarding tissue properties and underlying changes with diseases^33–37^. In a recent study using mouse models with varying melanin pigmentation levels, we showed that directional reflectivity (directional back-scattering) of the RPE layer when measured with a directional OCT (dOCT) is directly linked with the RPE melanin concentration^35^. This result showcased directional RPE scattering as a potential biomarker for various retinal diseases that induce alterations in the density of RPE melanosomes.

Similarly, short-wavelength fundus autofluorescence (SW-FAF) imaging (excitation ~ 488 nm and emission > 500 nm) has been widely used for monitoring lipofuscin accumulation in the RPE. SW-FAF imaging is generally performed with a confocal scanning laser ophthalmoscope (cSLO) since the confocal optics allow the acquisition of an AF signal from the RPE/Bruch’s membrane (RPE/BrM) complex while rejecting AF from anterior parts of an eye such as the cornea and lens. However, the accuracy of the intensity-based SW-FAF assessment is limited due to its dependence on multiple factors, such as the position of the focal plane in respect to the RPE/BrM complex, ocular transmission, data acquisition, gain, AD conversion, image processing, and variation between the systems^38,39^. Assessment based on the spectral profile, or shape, of the emission spectrum could overcome most of the above limitations with intensity-based SW-FAF and provide an improved characterization of lipofuscin and its changes.

While near-infrared fundus autofluorescence (NIR-FAF) imaging (excitation ~ 785 nm, emission > 810 nm) has been used to monitor changes in melanin in the outer retina^40,41^, it suffers from inherent issues with confocal axial resolution and overlapping strong NIR-FAF from choroidal melanosomes, making the distinction between RPE and choroidal melanosomes difficult to assess. The credibility of the assessment of the RPE melanosomes using NIR-FAF is further challenged by the contribution of lipofuscin to the NIR-FAF signal^42,43^. Therefore, measuring directional reflectance (dOCT), rather than NIR-FAF, and combining these data with SW-FAF signal (images and spectra) of RPE can provide critical complementary information regarding the changes of the melanosomes and lipofuscin in RPE.

In this paper, we report an in vivo multimodal investigation using directional back-scattering (directional reflectivity) and SW-FAF, that reveals changes in the density of lipofuscin granules and melanosomes in the RPE of 14-month-old *Abca4*^−/−^ mice (a model of Stargardt disease) relative to age-matched wild-type (WT) controls. The in vivo results were validated with ex vivo confocal imaging and electron microscopy from the same animals.

## Materials and methods

### Animal models and preparation for in vivo imaging

All animal procedures were performed with approval by the University of California, Davis Institutional Animal Care and Use Committee (IACUC). The study was conducted in accordance with all relevant guidelines and regulations, including the National Institute of Health (NIH), the Association for Research in Vision and Ophthalmology (ARVO), and Animal Research: Reporting of In Vivo Experiments (ARRIVE). A cohort of 14-month-old pigmented (agouti background) WT (129S1/SvlmJ) and age-matched *Abca4*^−/−^ (129S-Abca4^tm1Ght/J^) mice were used in this study. Both strains were purchased from the Jackson Laboratory (Sacramento, California, USA, strains #002448 and #023725).

### In vivo multimodal imaging

Both eyes of three WT and three *Abca4*^−/−^ mice were imaged for the in vivo studies; no animals, experimental units, or data points were excluded from analysis. During in vivo imaging, each mouse was placed on a micropositioner (Bioptigen, Durham NC, USA) that allowed rotational and translational adjustment of the mouse with respect to the SLO/OCT imaging system. During image and spectrum acquisition, mice were anesthetized with the inhalational anesthetic isoflurane (2% in O_2_). Pupils were dilated with 1% tropicamide and 2.5% phenylephrine (Akorn, Inc., Lake Forest, Illinois, USA), and the corneal surface was wetted with Gel Tears hypromellose gel (GenTeal Tears, Severe, Alcon, Texas, USA). The multimodal assessment of RPE based on dOCT and SLO-based AF were not made simultaneously, but rather with two separate custom imaging systems. To the best of our ability, the dOCT and SLO data were acquired from the same retinal eccentricity. The two imaging systems were located side-by-side in our lab, and we performed dOCT and AF imaging one after another under the same experimental conditions.

### In vivo assessment of the directional reflectivity

A swept-source -based dOCT system was used to measure the directional reflectivity of the RPE and other retinal layers in vivo as previously described^35^. In brief, the swept-source (Excelitas Technologies Corp., Waltham, Massachusetts, USA) used for OCT imaging has a center wavelength of ~ 1060 nm and bandwidth of ~ 100 nm. The comprehensive details of the assessment of the directional reflectivity of retinal layers with our dOCT system have been reported previously^35^. In OCT, an A-scan is a one-dimensional signal representing the depth-resolved reflectivity profile of a sample. Therefore, an A-scan provides information about the reflective and scattering properties of the sample as a function of depth. A B-scan, or cross-sectional image, is then produced by transversally scanning the beam across the sample and assembling a collection of neighboring A-scans. The directional reflectivity of the RPE/BrM complex and other layers was calculated from the dOCT B-scans. DOCT images of the retina were acquired by moving the position of the imaging beam with respect to the dilated mouse pupil using motorized linear stages. The lateral displacement of the OCT beam changes the angle of illumination of the retina, resulting in tilting of the dOCT images. The angle of incidence at each pupil position was calculated from the acquired B-scan after converting the image to a 1∶1 aspect ratio in axial and transversal directions. The entrance pupil position was shifted to a maximum of 800 μm on either side of the pupil center with a step of 100 μm in orthogonal directions horizontal (H) and vertical (V). Five closely spaced (2 μm separation) OCT B-scans were acquired (total separation between first and last B-scan of ∼8 μm) at each pupil position along the Y direction (orthogonal to laser scanning) and averaged. The length of all B-scans was a 20° field of view (FOV) which corresponds to approximately 680 μm in the X and Y direction (scanning) respectively. The averaged B-scans collected from each beam entry position were next tilt-corrected using affine transform and registered using ImageJ (Stack Reg)^44^. From each tilt-corrected B-scan, twenty well-aligned intensity A-scan profiles were selected near the center region and averaged for further analysis. To ensure that the variation in directional reflectivity of the RPE/BrM complex between strains was induced only by the changes in melanosome density, directional reflectivity of other melanin-free retinal layers, including the inner limiting membrane (ILM), external limiting membrane (ELM), and inner-outer segment (IS/OS) junction, were also measured and compared. The directional reflectivity of these layers was similarly calculated from averaged A-scan profiles. The measured values of reflectivity as a function of angle of incidence were fitted with a Gaussian function provided in Eq. (1).

1 $$I\left( x \right) = a_{0} + a_{1} *10^{{ - \rho [\left( {x - x_{0} } \right)^{2} + \left( {y - y_{0} } \right)^{2} ]}}$$

where $a_{0}$ and $a_{1}$ represent the nondirectional (background) and directional components of retina reflection, respectively, ρ is the directionality parameter, and $x_{0}$ and $y_{0}$ represent the geometric center of the pupil of the dilated eye. The sensitivity of the directional reflection of retinal layers was quantified using a directionality parameter (ρ) from Eq. (1). The parameter ρ is inversely proportional to full-width-at-half-maximum (FWHM) of the fitted Gaussian function. Therefore, a wider profile (larger FWHM) gives a small value of ρ, and vice versa.

### In vivo autofluorescence measurement

The SW-FAF images and emission spectra were acquired in vivo using a custom-built SLO integrated with a spectrometer (QE 65,000, Ocean Optics, Florida, USA)^45^. The SLO system allows the acquisition of FAF images and spectra from any discrete region of interest of the fundus. The fundus images and emission spectra were acquired using 488 nm excitation with a power of 105 μW at the mouse pupil. A high-pass spectral filter with a cut-off wavelength of 503 nm (Semrock, New York, USA) was used as an emission filter. The AF spectra were measured from the same location where the dOCT imaging was performed. The weak FAF signals were amplified with a photomultiplier tube and a gain controller (Hamamatsu H7422-50, Japan). The integration time for the spectrometer was set to 2.5 s for all spectral acquisitions. Stray fluorescence from the optical elements and dark-leakage current from the detector were recorded separately (without the mouse eye) and subtracted from the fundus AF spectra measured from the mouse. During the collection of fluorescence emission spectra, the fundus regions containing blood vessels and optic nerve head were excluded to ensure that emission was solely contributed by the fluorophores in the RPE.

### Preparation of retina flat-mount for ex vivo confocal studies

After completion of in vivo imaging, mice were euthanized by CO_2_ narcosis, and one eye was enucleated. The enucleated eye was placed in a petri dish containing an ice-cold solution of DMEM (Dulbecco’s Modified Eagle Medium, with high glucose and no phenol red, Thermo Fisher 21-063-029) supplemented with 10% fetal bovine serum (FBS, Corning 35010CV) and NucBlue nuclear probe (2 drops per ml of solution, Molecular Probes Thermo Fisher R37605). The eyecup was shaped similar to a four-leaf clover after removing the anterior segment, lens, muscles and connective tissues on the back of the sclera, and the optic nerve. The retina was then gently peeled off from the eyecup, exposing the RPE layer which remained attached to the choroid and sclera. This latter preparation was placed scleral side down onto a translucent membrane (Whatman 110614). The flat-mount tissue on the membrane was transferred to a Lab-Tek II Chambered Coverglass (Thermo Fisher 155379PK), and placed RPE-side down directly on the cover glass. A custom weighted mesh was placed on top of the membrane, and then fresh, ice-cold DMEM supplemented with 10% FBS was added to the chambered coverglass. The chambered coverglass was then placed on the stage of the Nikon A1 confocal microscope fitted with a LiveCell Stage Top Incubation System (Pathology Devices). For the duration of imaging, the LiveCell system was set to maintain temperature at 36 °C, humidity at 60%, and CO_2_ at 10%.

### Ex vivo confocal imaging of the flat-mounted RPE

The high-resolution in situ confocal images of the flat-mounted RPE of both strains were acquired with a Nikon A1 confocal microscope (New York, USA) as previously described^46^. Both confocal fluorescence images and emission spectra were acquired at 488 nm excitation. The confocal images were acquired with and without an emission filter in the detection path. A 60 X objective lens (Nikon, Plan Apo VC 60X, Water Immersion) with a numerical aperture of 1.2 was used to acquire confocal image stacks. Twenty-seven confocal planes were acquired from RPE tissue in the Z-direction (depth) with a step size of 0.5 μm. NIS-Elements AR processing software was used for all processing and analysis of the confocal images and emission spectra. 3D image stacks were deconvolved with the confocal point spread function to correct confocal artifacts and chromatic aberration. The ‘spectral unmixing’ feature of the software was used for the segmentation and quantification of individual fluorophores within the RPE volume.

### Ex vivo imaging with electron microscopy

Electron microscopy (EM) images of the RPE were acquired with a transmission electron microscope (TEM). Euthanized mice were transcardially perfused with 2% paraformaldehyde, 2% glutaraldehyde, and 0.05% calcium chloride in 50 mM MOPS, pH 7.4^47^. The eye was enucleated, and the cornea and lens were removed. The posterior eye cup was post-fixed 2 h then rinsed with PBS. The tissue was blocked, then rinsed in 0.1 M sodium phosphate buffer, stained with 1% osmium tetroxide, rinsed in dH20, and dehydrated with an increasing series of ethanol. Blocked tissue was placed in a pre-inflitrate of 50% propylene oxide and 50% PolyBed resin overnight, then infiltrated with 100% PolyBed resin and polymerized in fresh resin. Thin sections (80–90 nm) were cut on an ultramicrotome, collected on copper slot grids, and stained with 4% uranyl acetate and 0.3% lead citrate in 0.1 N sodium hydroxide and examined with a Talos L120C TEM. The morphological features of melanosomes, lipofuscin, and melanolipofuscin granules were identified and classified from EM images using the “ParticlePicker” plugin available with ImageJ software^48,49^. For the quantification, each granule was manually marked with colored dots in a new map based on their actual center location in the EM image using a custom MATLAB script.

### Statistical analysis

PRISM (Version 7.02; GraphPad Software, La Jolla, CA, USA) was used for statistical analysis. Results are presented as mean with standard error (SE) of three mice per group unless otherwise specified. The null hypothesis was that the groups were not statistically different. Two-group comparisons were performed with ANOVA, and a p-value of 0.05 or less was considered statistically significant.

## Results

### Directional scattering from the RPE layer

**Figure 1:**
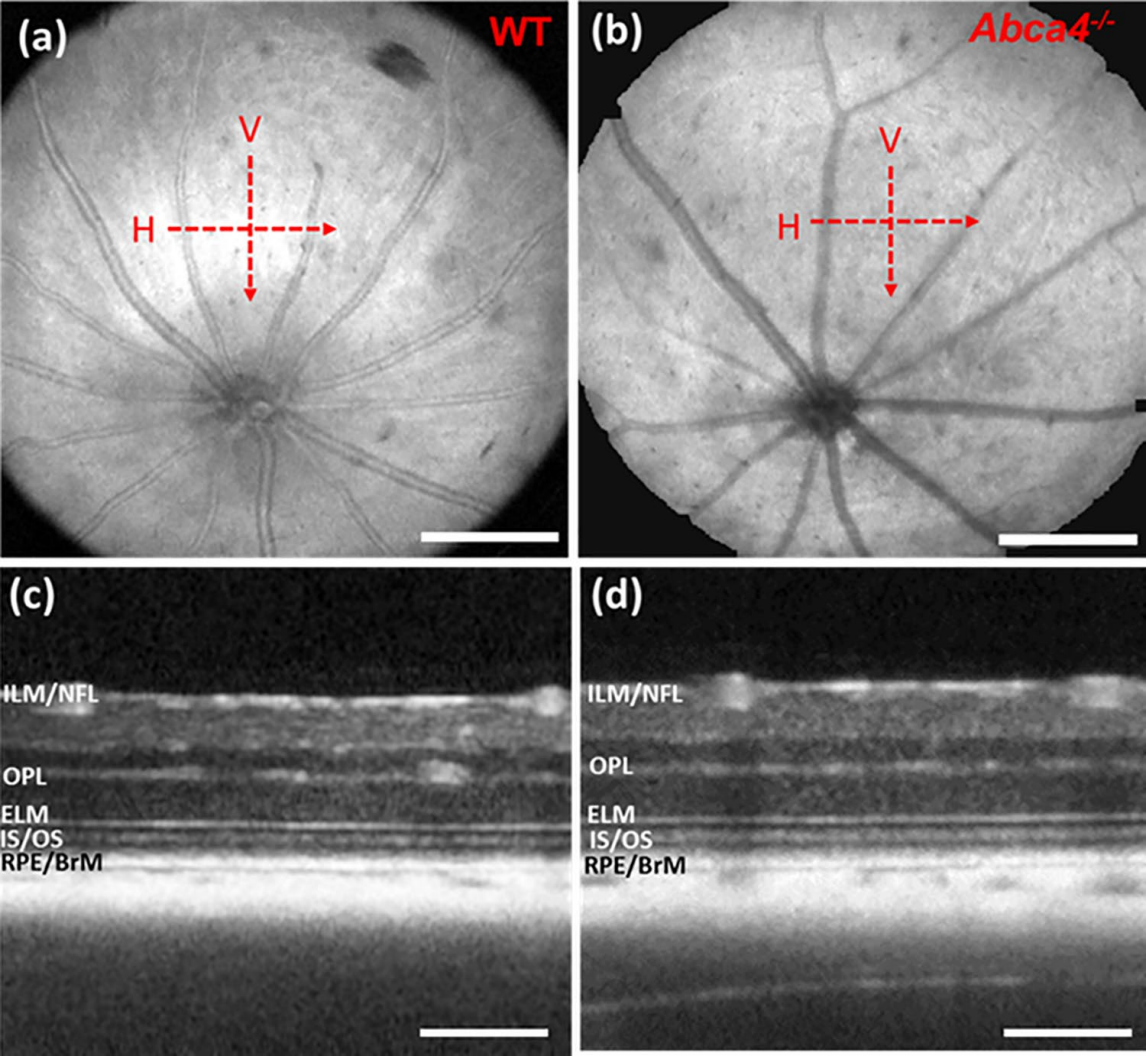
Representative *en face* OCT fundus and B-scan images. (**a**) *En face* fundus image of a WT mouse. (**b**) *En face* fundus image of an *Abca4*^−/−^ mouse. Red dashed arrows show the location and direction of OCT scanning in horizontal (H) and vertical (V) directions. (**c**) Averaged B-scan acquired (zero-degree incident angle) from a WT mouse along the H direction. (**d**) Averaged B-scan acquired (zero-degree incident angle) from an *Abca4*^−/−^ mouse along the H direction. Scale bar (**a**,**b**): 400 μm; scale bar (**c**,**d**): 200 μm.

Directional retinal OCT imaging was performed in both mouse strains at a similar retinal eccentricity (distance from ONH) as depicted in *en face* OCT fundus images shown in Fig. 1a,b. The SSOCT system, which has an axial resolution of ~ 7.5 μm in air, visualized major retinal boundaries and layers such as ILM/NFL, outer plexiform layer (OPL), ELM, IS/OS junction, and RPE/BrM (Fig. 1c,d). Imaging distal tips of the photoreceptor outer segments was not possible with this system due to the limited axial resolution.

**Figure 2:**
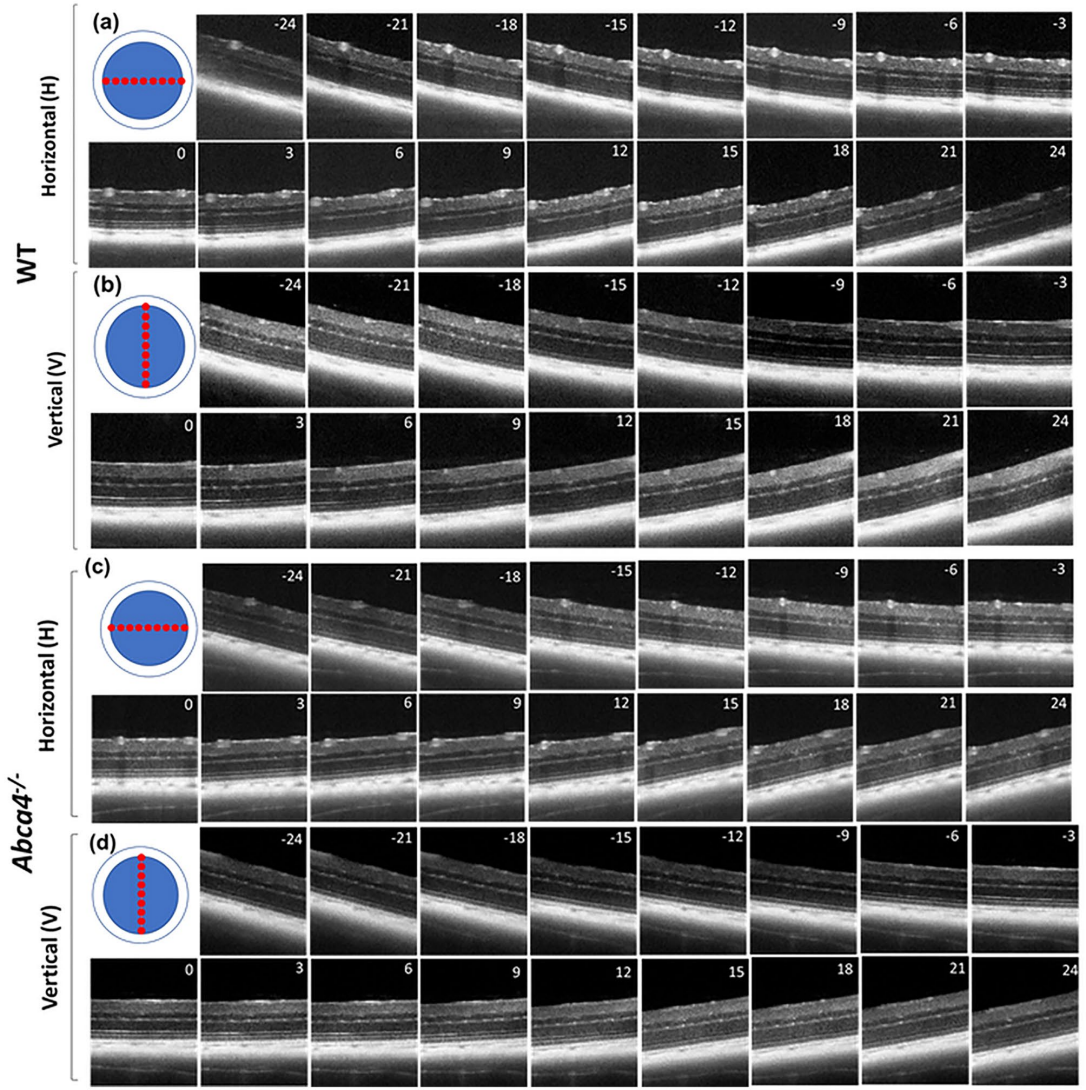
Averaged representative directional OCT B-scans (log scale) acquired from a WT and an *Abca4*^−/−^ mouse. (**a**) B-scans from a WT mouse corresponding to 17 beam entry positions along the H direction. (**b**) B-scans from a WT mouse corresponding to 17 beam entry positions along the V direction. (**c**) B-scans from an *Abca4*^−/−^ mouse corresponding to 17 beam entry positions along the H direction. (**d**) B-scans from an *Abca4*^−/−^ mouse corresponding to 17 beam entry positions along the V direction. The number provided in each B-scan represents the incidence angle of retinal illumination.

DOCT images of retinas from both mouse strains were acquired by varying beam entry positions at the pupil (varying angles of incidence) on the retina along vertical (V) and horizontal (H) directions (Fig. 2). This movement of the pupil entry position creates tilt in the B-scans. The average tilt of the B-scan was measured to be approximately 3° for every 100 µm of lateral displacement at the entrance pupil position. Acquired dOCT images showed that the reflectivity of many retinal layers depends on the angle of incidence, and visibility of these structures varies as a function of the angle of illumination.

**Figure 3:**
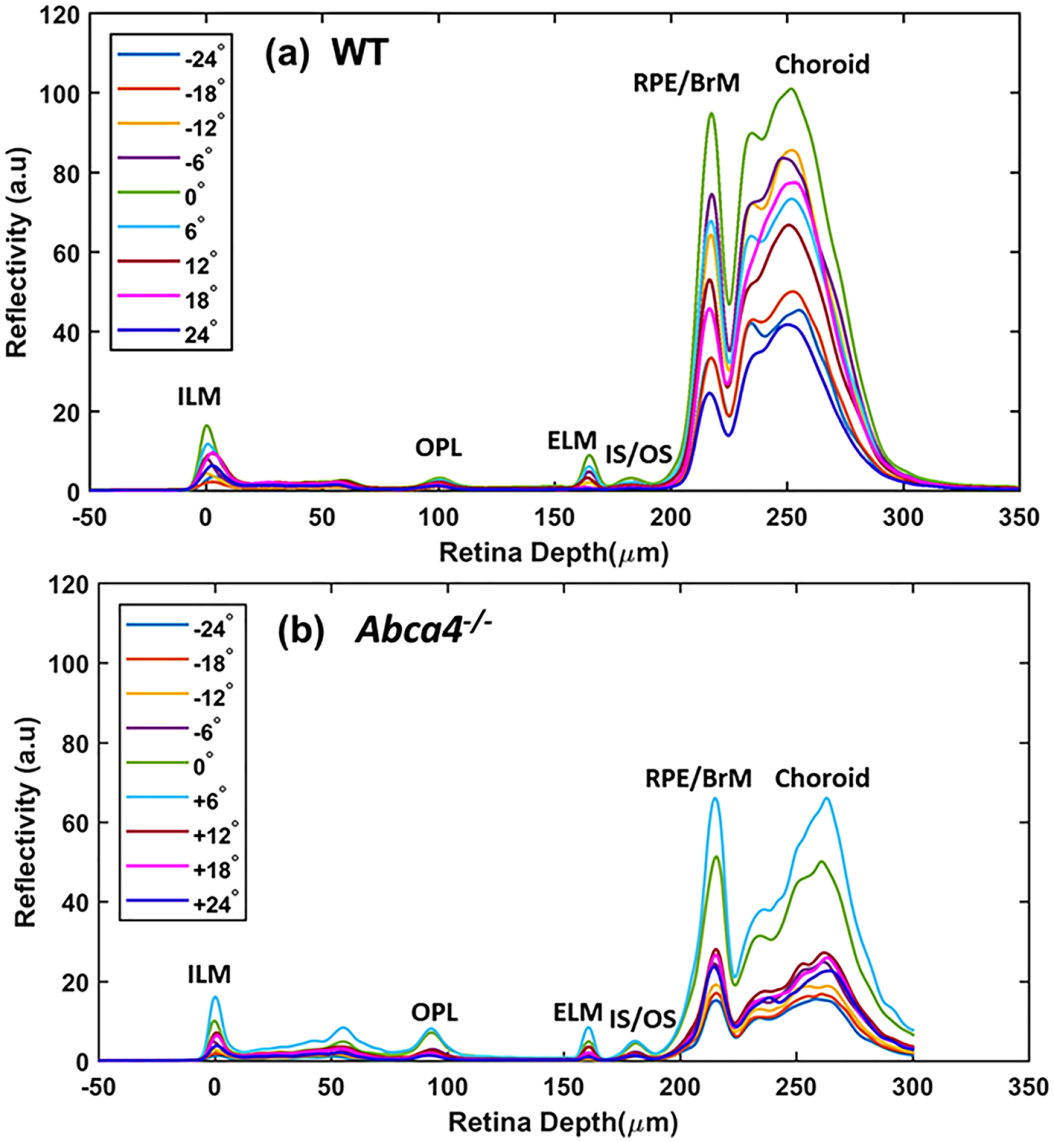
Representative average A-scans (linear scale) obtained for different angles of incidence from WT and *Abca4*^−/−^ mice. (**a**) Average A-scans obtained along the H direction from a WT mouse. (**b**) Average A-scans obtained along the H direction from an *Abca4*^−/−^ mouse.

The reflectivity of the retinal layers was quantitatively measured from the averaged A-scan profile extracted from the dOCT images (Fig. 3). The multiple peaks that appeared in the A-scan profiles provided the position and magnitude of the reflectivity of hyperreflective layers such as ILM, OPL, ELM, IS/OS junction, and RPE/BrM complex. The RPE/BrM complex showed the highest peak, or reflectivity, among all layers and varied the most with angle of incidence.

**Figure 4:**
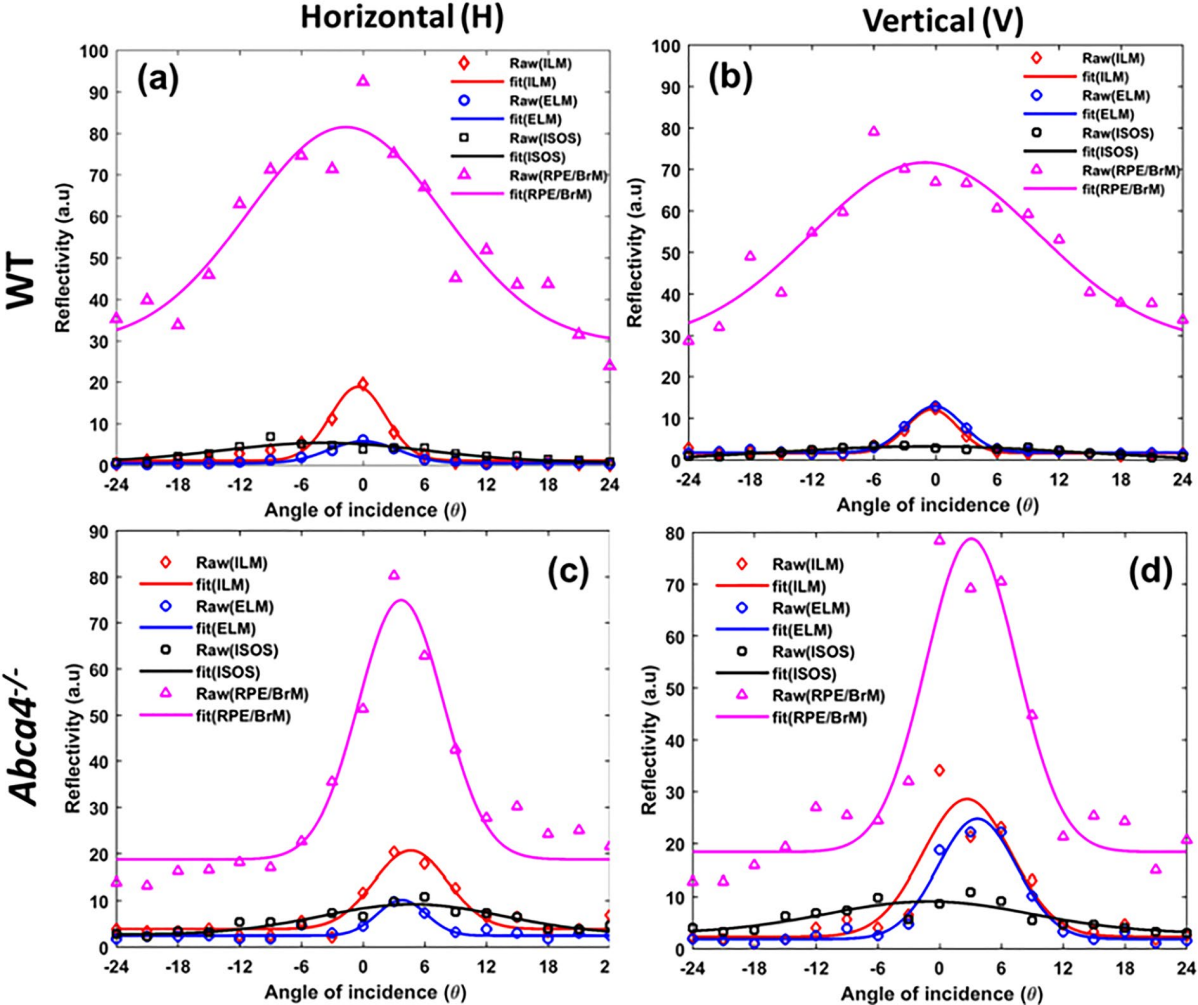
Representative Gaussian fits to the dOCT based layer-specific and incident angle-dependent reflectivity profiles for WT (**a**,**b**) and *Abca4*^−/−^ mice (**c**,**d**). (**a**,**c**) reflectance of different retina layers as a function of angle of incidence measured along the H direction. (**b**,**d**) reflectance of different retina layers as a function of angle of incidence measured along the V direction.

The incident angle-dependent reflectivity of the RPE/BrM complex and three other layers (ILM, ELM, and IS/OS) of both strains were measured from A-scan profiles and fitted with a Gaussian function (Fig. 4). The directionality parameter (ρ) of different retinal layers was obtained from a Gaussian function using Eq. (1). Table 1 lists the mean and standard error of the directionality parameter measured for different retinal layers in both strains. A large value of the directionality parameter represents a narrow reflectivity profile, which implies a highly directional scattering. Consistent with our previous observations, scattering from the ILM/NFL and ELM was highly directional, whereas the IS/OS junction in both strains exhibited a wider reflectivity profile (Fig. 4a,b). Furthermore, the directionality parameters measured for the ILM/NFL, ELM, and IS/OS, which all lack melanosomes, are similar and do not statistically differ between the two mouse strains (all p-values > 0.05). However, the directional reflectivity profile of the RPE/BrM complexes of the two strains are significantly different (p = 0.01). The Gaussian profile obtained from WT mice is wider than that obtained from *Abca4*^−/−^ mice: the average directionality parameter of the RPE/BrM complex measured for *Abca4*^−/−^ mice is nearly fourfold higher than for WT.

**Table 1:** Mean values of directionality (ρ) with standard error.

Layers	*Abca4*^−/−^		WT	
	H	V	H	V
ILM/NFL	0.240 (± 0.025)	0.232 (± 0.022)	0.265 (± 0.018)	0.274 (± 0.014)
ELM	0.222 (± 0.025)	0.219 (± 0.057)	0.247 (± 0.015)	0.263 (± 0.011)
IS/OS	0.018 (± 0.010)	0.023 (± 0.004)	0.026 (± 0.005)	0.039 (± 0.008)
RPE/BrM	0.171 (± 0.013)	0.184 (± 0.025)	0.041 (± 0.015)	0.052 (± 0.012)

### Fundus AF images and emission spectra

**Figure 5:**
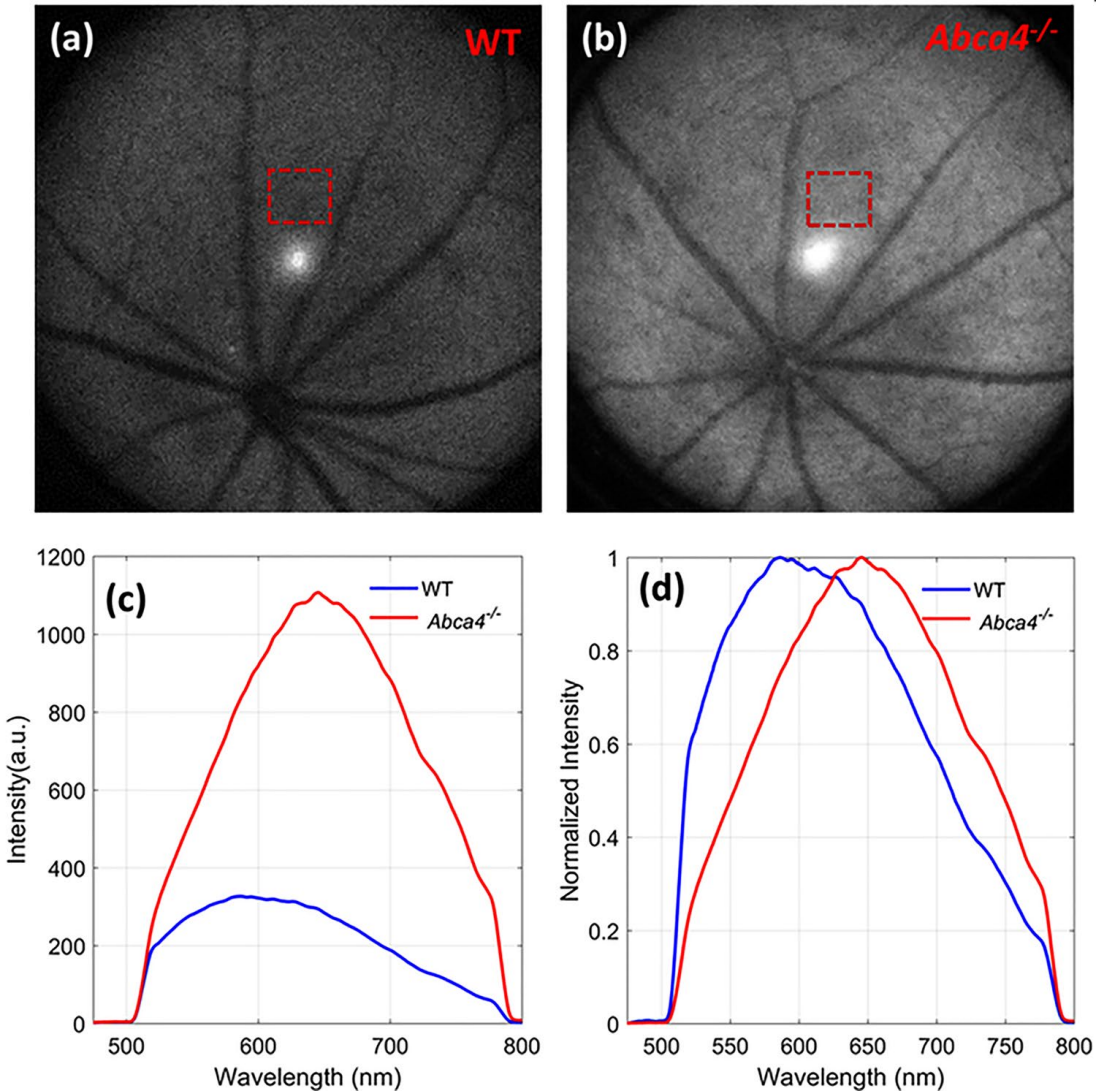
In vivo FAF images and emission spectra acquired with 488 nm excitation. Representative FAF images from (**a**) a WT and (**b**) an *Abca4*^−/−^ mouse. (**c**) Absolute emission spectra measured from a WT control and an *Abca4*^−/−^ mouse. (**d**) Normalized emission spectra from (**c**). Dashed red rectangles in (**a**,**b**) show the locations where AF spectra were measured. The sharp decline in fluorescence in (**c**,**d**) at about 510 nm and 780 nm are due to dichroic cutoff filter.

FAF images and emission spectra acquired in vivo from WT and *Abca4*^−/−^ mice are shown in Fig. 5. Figure 5a,b are the representative FAF images obtained from WT and *Abca4*^−/−^ mice, respectively. Figure 5c,d represent the in vivo recorded AF spectra from the fundus area (shown in red dashed boxes in a, b) of the WT and *Abca4*^−/−^, respectively. There was notably increased intensity of the AF from *Abca4*^−/−^ mice compared to WT, approximately 3.4-fold higher. Additionally, a significant “red” shift in the fluorescence emission spectrum was observed in the *Abca4*^−/−^ relative to the WT.

### Ex vivo confocal fluorescence images

**Figure 6:**
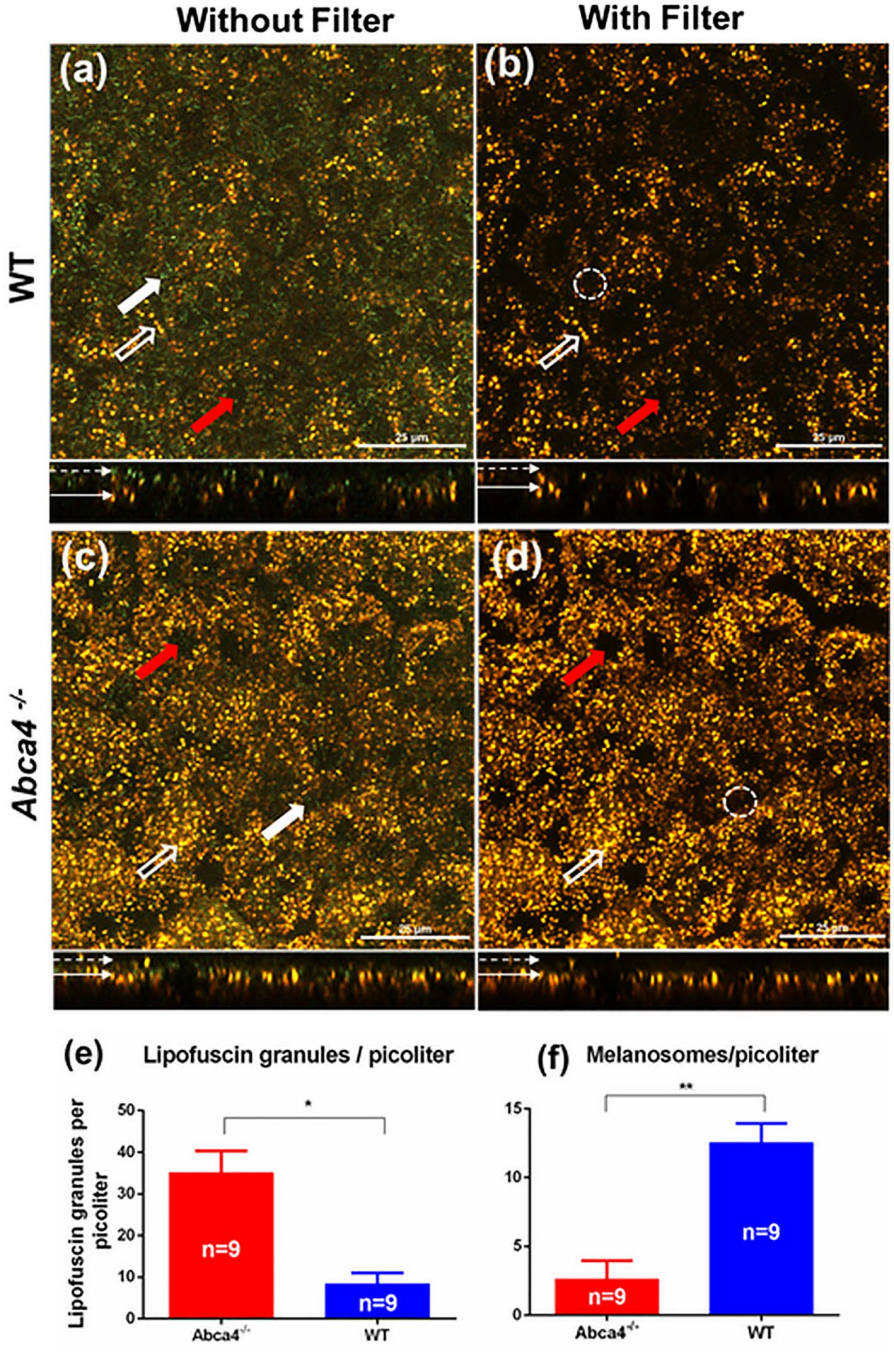
Confocal images of flat-mounted RPE from WT and *Abca4*^−/−^mice. (**a**,**c**) Confocal images of the RPE cell mosaic acquired without emission filter. (**b**,**d**) Confocal images of RPE cell mosaic acquired with emission filter. (**e**,**f**) bar graphs showing the density of lipofuscin and melanosomes as number of granules per picoliter. The average number of lipofuscin granules and melanosomes were calculated from 9 confocal volumes per strain. White solid arrows on top panels indicate representative melanosomes, whereas the hollow arrows indicate representative lipofuscin granules. The red arrows indicate representative nuclei. White dashed-circles in (**b**,**d**) indicate the absesnce of melanosomes in the same region indicated by thick white arrows in (**a**,**c**). The melanosomes are not visible in (**b**,**d**) because the scattered light from the melanosomes is fully blocked by the fluorescense emission filter. The bottom panels in (**a**–**d**) show side views of the RPE volume. The dotted arrows in the bottom panel show the apical side of the RPE cells, whereas the solid arrows indicate the basal side of the RPE. (*p < 0.001,**p < 0.001). Scale bars: 25 μm.

High-resolution confocal images of live RPE flat-mounts from WT and *Abca4*^−/−^ mice were acquired with 488 nm excitation (Fig. 6). Confocal images of the RPE flat-mounts were obtained with and without an emission filter in the microscope detection path. Figure 6a,c show representative confocal images (depth projected) acquired from WT and *Abca4*^−/−^ mice, respectively, without an emission filter. Lack of an emission filter permitted the measurement of back-scattered light from the RPE, resulting in the visualization of both green-colored spindle-shaped melanosomes (thick white solid arrows) and golden-yellow-colored spherical lipofuscin granules (white hollow arrows)^46^. Figure 6b,d represent the confocal images acquired with an emission filter inserted in the detection channel of the microscope. The melanosomes are not visible in these images because the scattered light from the melanosomes is fully blocked by the emission filter, so only AF signals reach the spectral detector. Consistent with our previous observations, melanosomes were predominantly located at the apical side of the RPE (dashed white arrow) and lipofuscin granules mainly occupied the basal region of the RPE (solid thin arrow)^46^.

Qualitative and quantitative analyses revealed differences in the density of the melanosomes and lipofuscin granules (number of granules per picolitre) between *Abca4*^−/−^ and WT RPE cells. There is a reduction in the density of melanosomes in the RPEs of *Abca4*^−/−^ relative to WT mice. In contrast, the number of lipofuscin granules in *Abca4*^−/−^ mice is significantly greater than in the WT. The average density of the lipofuscin granules and melanosomes were calculated from the RPE confocal volumes (Fig. 6e,f). The density of lipofuscin granules is approximately 4.2 times higher in *Abca4*^−/−^ than in WT mice, whereas the density of melanosomes declined in *Abca4*^−/−^ mice by a factor of approximately 4.7 compared to WT mice.

**Figure 7:**
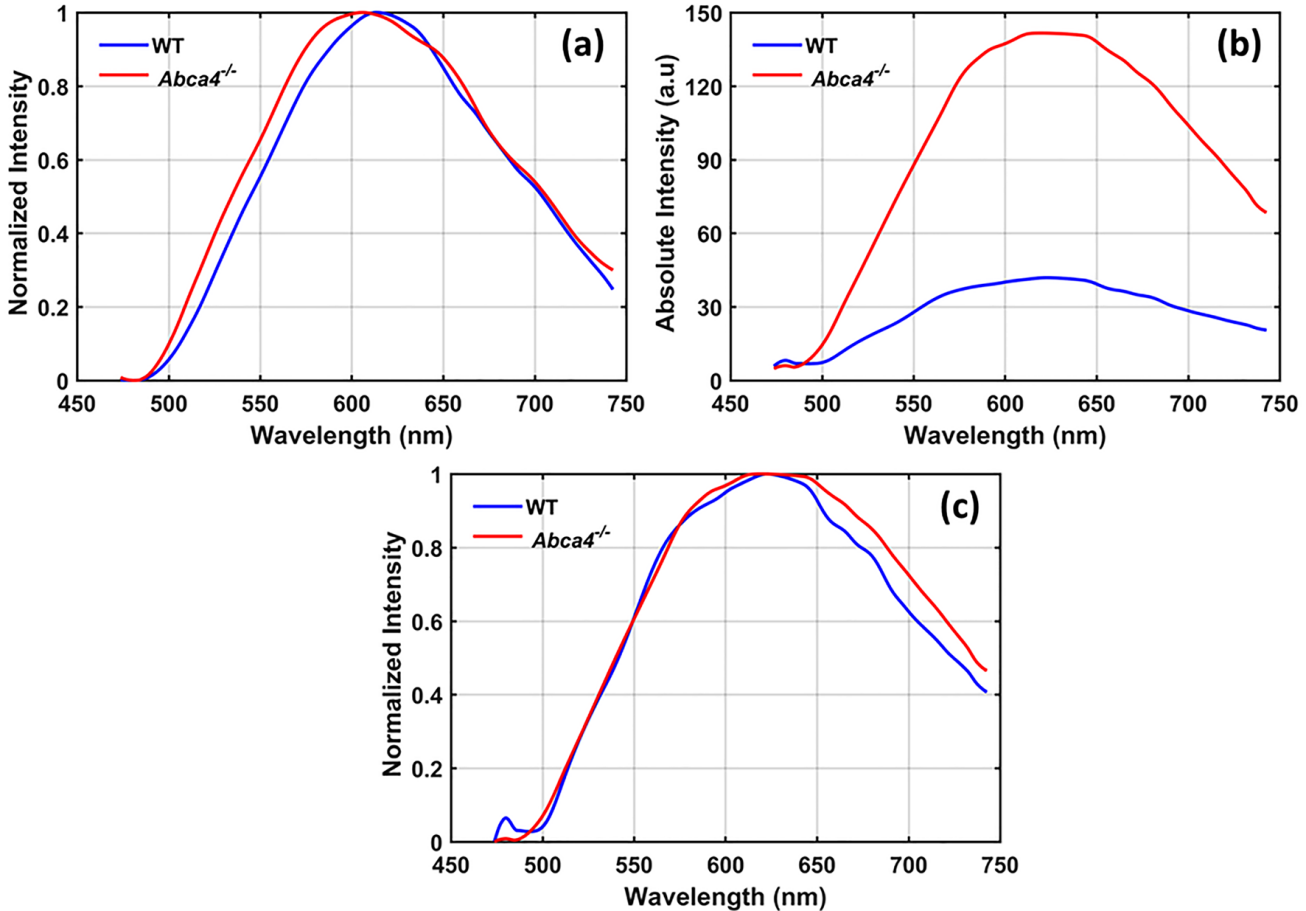
AF emission spectra obtained with ex vivo confocal imaging for an excitation wavelength of 488 nm. (**a**) Average emission spectra acquired from 50 lipofuscin granules (spectra were normalized to the emission peak). (**b**) Average emission spectra measured from the whole confocal volume with identical excitation power. (**c**) Normalized spectra from (**b**).

The normalized average emission spectra from 50 lipofuscin granules from both strains were extracted from confocal data (Fig. 7a). No significant change in the emission peak or spectrum shape was found between the mouse strains. The average AF emission spectra from the entire confocal volume were acquired, and the intensity of the emission spectrum from *Abca4*^−/−^ RPEs was elevated by a factor of ~ 3.9 relative to WT (Fig. 7b). The normalized spectra (Fig. 7c) from the confocal volume revealed that the peak emission wavelength and spectrum shape do not differ materially between the two strains.

### Electron microscopy images of RPE

**Figure 8:**
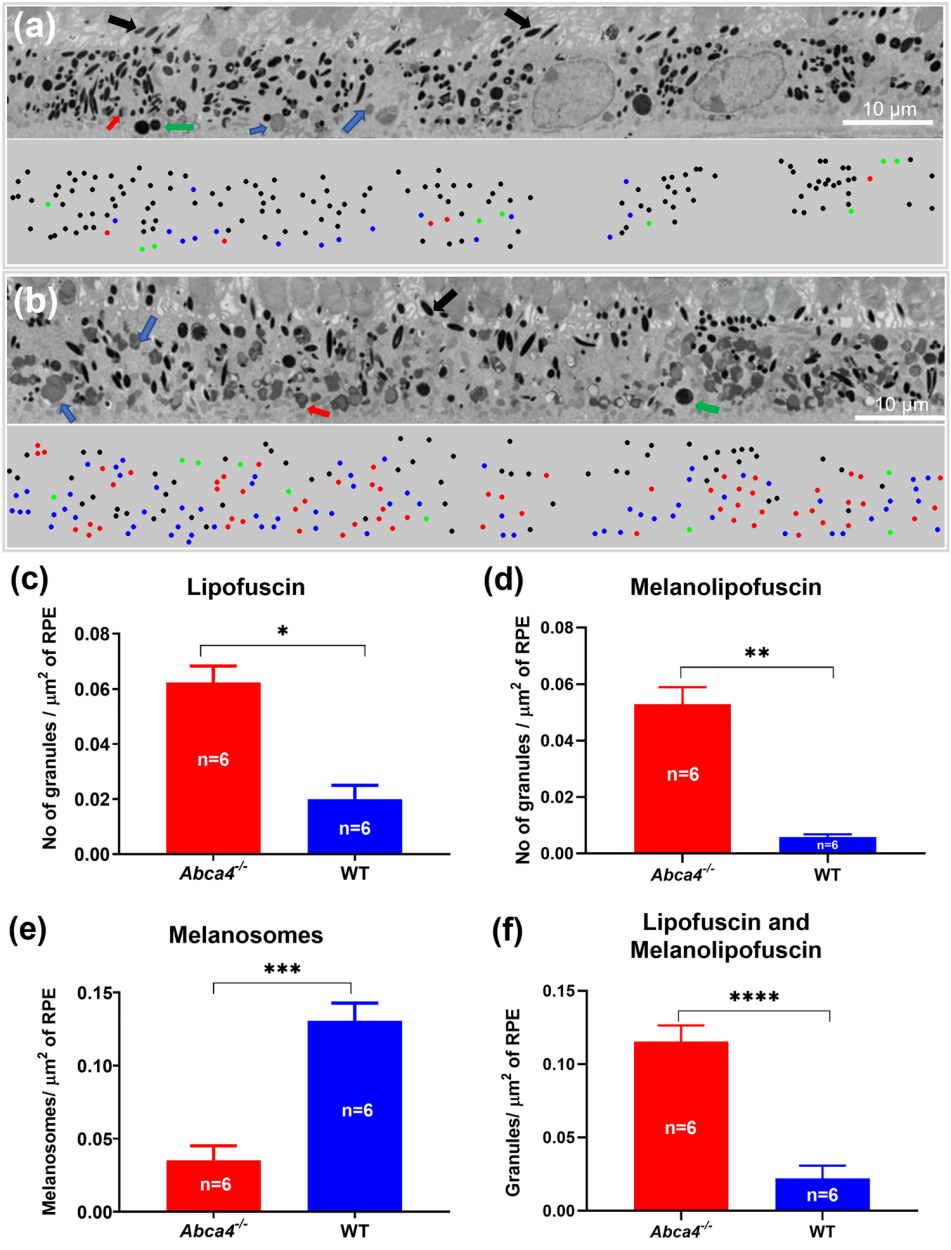
Representative EM images showing cross sections of the RPE cell layer and RPE granules. (**a**) The RPE cell layer of a WT mouse. (**b**) The RPE cell layer of an *Abca4*^−/−^ mouse. The bottom panels of (**a**,**b**) are the color-coded classified granules showing their locations. Number of granules per μm^2^ calculated for (**c**) lipofuscin, (**d**) melanolipofuscin, (**e**) melnaosomes, and (**f**) combined lipofuscin and melanolipofuscin. The values shown are means and standard error. Six images were quantified per strain (one eye each from 2 animals per group were analyzed and 3 images per eye were counted). Black arrows and dots: melanosomes; Blue arrows and dots: lipofuscin; Green arrows and dots: phagocytosed outer segments; red arrows and dots: melanolipofuscin, Scale bars: 10 μm. (*p < 0.001, **p < 0.01, ***p < 0.001, ***p < 0.001).

Electron microscopy images of the RPE cell layer from WT and *Abca4*^−/−^ mice are shown in Fig. 8a,b, respectively. These axial cross-section EM images contain all of the major granule types of RPE cells: melanosomes, lipofuscin, melanolipofuscin, and phagocytosed outer segments^50^. Notable differences between strains were found regarding the density of these RPE granules. The bottom panel of each EM image represents the mapping of the locations of each type of RPE granule. The densities (mean number of granules per µm^2^ of imaged RPE) of lipofuscin and melanolipofuscin granules in the RPE cells of WT were 0.020 ± 0.009 and 0.006 ± 0.001 respectively. For calculating the area of RPE from EM images shown in Fig. 8a,b, any area that included photoreceptor outer segments was excluded. In contrast, the densities of lipofuscin and melanolipofuscin granules in *Abca4*^−/−^ RPE cells were 0.062 ± 0.011 and 0.053 ± 0.006, respectively, an increase compared to WT (Fig. 8c,d). The density of melanosomes in WT and *Abca4*^−/−^ RPE cells were found to be 0.131 ± 0.064 and 0.035 ± 0.018, respectively (Fig. 8e). Overall, there was a 4.7-fold increase in the number of lipofuscin/melanolipofuscin granules in *Abca4*^−/−^ relative to WT RPE cells, and a 3.7-fold decrease in the number of melanosomes in *Abca4*^−/−^ RPE cells as compared to WT (Fig. 8f).

## Discussion

Measurements with the dOCT system revealed strain-specific variation in the directionality of scattering from the RPE/BrM complex and other retinal layers while melanin-free layers (ILM/NFL, ELM, and IS/OS) exhibited essentially identical directional scattering properties across the two strains. However, a striking difference was observed in the directional reflectivity of the RPE/BrM complex (Fig. 4). In particular, the RPE/BrM complex of *Abca4*^−/−^ showed an increased directionality relative to the WT controls (Table 1). An increased directionality from RPE can be linked to a decreased melanin concentration in RPE cells^35^. Melanosomes are predominantly located in the apical side of the RPE cell and are the dominant light scatters (Mie scattering) in the RPE cells. The nature of scattering (angular distribution) from melanosomes depends on their shape and size, which determine the diffraction parameter^35,51,52^. In *Abca4*^−/−^ mice, the density of RPE melanosomes is significantly reduced and the density of melanolipofuscin granules is significantly increased (Fig. 8). Melanolipofuscin combines features of lipofuscin and melanosomes, containing an electron-dense spindle, sometimes ovoid and fibrillar, within a larger granule^10,49^. Relatively large sized or clustered form (large effective size) of the melanolipofuscin granules in *Abca4*^−/−^ could cause the forward scattering dominant for these granules^10,23,49^. This allows the forward propagation of incident light, resulting in a highly directional back-scattering from BrM^35^. Overall, directional scattering assessment indicated that there is a relative deficit in melanosome density in the RPE cells of *Abca4*^−/−^ mice relative to the WT.

In vivo SW*-*FAF images from *Abca4*^−/−^ mice showed increased fluorescence intensity relative to WT (Fig. 5). Both in vivo FAF images and spectral measurements confirmed that there is an increased accumulation of lipofuscin in the RPE cells of *Abca4*^−/−^ mice. This is further confirmed by the qualitative and quantitative analysis of the ex vivo confocal images, which showed the excess accumulation of lipofuscin granules in the RPE of *Abca4*^−/−^ mice (Fig. 6b,d). The peak emission wavelength measured in vivo (~ 590 nm for WT and ~ 645 nm for *Abca4*^−/−^) was in good agreement with the ex vivo results (~ 635 nm). These peak emission wavelengths are consistent with the peak emission wavelength reported for lipofuscin from an *Abca4*^−/−^ mouse and human RPE cells^53,54^. Notably, the increase in SW-FAF from *Abca4*^−/−^ in vivo relative to WT (~ 3.4) is nearly the same as that seen in the ex vivo spectra (~ 3.9).

Comparison of confocal images acquired with and without an emission filter, to block backscattered light from the 488 nm excitation, confirmed the non-fluorescent characteristics of melanosomes for 488 nm excitation and identified lipofuscin granules as the major source of FAF. There was an increase in the density of lipofuscin granules by a factor of ~ 4.2 in *Abca4*^−/−^ relative to WT RPEs (Fig. 6e,f). It is noteworthy that this increase in the density of lipofuscin in *Abca4*^−/−^ is correlated well with the increase in AF levels obtained from both in vivo and ex vivo spectra. The AF spectra measured in vivo revealed a shift in the emission peak between WT and *Abca4*^−/−^. However, no such shift was observed in the ex vivo spectra measured from confocal images. This shift observed in in vivo spectra could be caused by the influence of other AF sources, or by differential spectral filtering of the backscattered light, a matter requiring further investigation.

Although confocal imaging was able to distinguish lipofuscin granules from melanosomes, it was not possible to discern a distinct population of melanolipofuscin granules. This might be due to the similar emission spectrum of the melanolipofuscin and lipofuscin granules^55,56^. Nonetheless, EM images provided visualization of distinct melanosomes, lipofuscin, and melanolipofuscin in the RPE of both strains. However, the actual sizes of the granules could not be accessed accurately with cross-sectional EM scans, and a three-dimension (3D) EM may be required for this purpose^49^. Consistent with the confocal imaging, EM images confirmed the presence of excess lipofuscin and melanolipofuscin granules in the RPE of *Abca4*^−/−^ mice relative to WT (Fig. 8c,d). The combined density of lipofuscin and melanolipofuscin quantified from EM images of *Abca4*^−/−^ RPEs is approximately 4.7-fold greater than WT (Fig. 8f). This increase agrees with the change in total number of AF granules (lipofuscin granules and melanolipofuscin) quantified from ex vivo confocal images, approximately 4.2-fold greater in *Abca4*^−/−^ RPEs than WT. EM images also showed a reduction in melanosomes in *Abca4*^−/−^ RPEs by a factor of 3.7, close to the factor of 4.7 obtained from ex vivo confocal images. Additionally, an increased directionality coefficient (ρ) of back-scattering in *Abca4*^−/−^ compared to WT mice by a factor of 4 suggests this coefficient is directly linked to the absolute changes in melanosome density. Thus, the directionality measure could serve as a possible non-invasive biomarker of RPE melanin concentrations in clinical settings. The quantitative assessment of RPE melanosomes from the EM images is in good agreement with both the in vivo and ex vivo confocal results. Given that melanosomes are not produced during adulthood, we hypothesize that the deficit in melanosome numbers in *Abca4*^−/−^ mice might arise from the formation of melanolipofuscin from the original (and possibly damaged) melanosomes in the presence of a high density of lipofuscin granules.

## Conclusion

In summary, we have demonstrated the potential of an in vivo multimodal investigation based on directional scattering and fluorescence measurements for studying disease-related pigmentary changes in the RPE. Disease-related changes in the major RPE pigment granules (melanosomes and lipofuscin) can be identified using a directional OCT and SW-AF-SLO system. The change in directional reflectance of the RPE measured with the dOCT system has been linked to the melanosome concentration in RPE cells. This dOCT based directional approach has an advantage over the conventional NIR-AF based imaging in that the change in RPE melanosomes can be investigated without any influence of choroidal melanosomes and lipofuscin. In vivo SW-AF images and emission spectra have been shown to be a reliable way of monitoring changes in lipofuscin density. The changes in melanosomes and lipofuscin granules measured in vivo correlated with the sub-cellular resolution ex vivo investigations performed with confocal fluorescence microscopy and electron microscopy that provided an enhanced visualization and quantification of each type of pigmented granule and their spectra (confocal). Overall, the measurements made in vivo correlated well with the ex vivo findings, highlighting the potential of a combined in vivo assessment combining directional scattering and SW-FAF spectra for longitudinal noninvasive monitoring of the changes in RPE melanosomes and lipofuscin granules during normal aging and the progression of outer retinal diseases. Finally, the clinical utility of this multimodal approach is clear, as these measurements can be easily done with current clinical OCT and SW-AF-SLO systems (combined or in separate instruments) without much modification of hardware or acquisition procedures.

## Data Availability

Images and datasets generated in this study are available from the corresponding author on reasonable request.
